# Epidemiological Characteristics and Co‐Detection Patterns of Rhinovirus (A/B/C) and Influenza A Virus in a Regional Respiratory Cohort in Hunan, China

**DOI:** 10.1111/crj.70184

**Published:** 2026-05-12

**Authors:** Feicheng Yang, Ping Bai, Jiaxuan Zhang, Juqi Peng, Qing Hu, Yanchun Li

**Affiliations:** ^1^ Department of Pathology Hunan Provincial People's Hospital (The First Affiliated Hospital of Hunan Normal University) Changsha Hunan China; ^2^ Medical College Hunan Normal University Changsha Hunan China

**Keywords:** coinfection, epidemiology, influenza A virus, rhinovirus, viral interference

## Abstract

**Objective:**

To characterize the epidemiological profile, coinfection spectrum, and virus–virus association patterns between rhinovirus (RV; A/B/C) and influenza A virus (IAV).

**Methods:**

We analyzed 8928 respiratory samples collected from April 2023 to December 2025. A quality‐controlled RV analytic cohort (*n* = 386) and an IAV‐positive cohort (*n* = 422) were used for subtype distribution, coinfection profiling, and observed‐versus‐expected co‐occurrence analysis. Associations were evaluated with Fisher's exact test or chi‐square test, with odds ratios (ORs) and 95% confidence intervals (CIs).

**Results:**

In the RV cohort, subtype A was dominant (235/386, 60.9%), followed by C (118/386, 30.6%) and B (33/386, 8.5%); mixed subtypes accounted for 1.6% (6/386). Coinfection was common (282/386, 73.1%), and IAV was detected in 14/386 RV‐positive samples. Co‐occurrence analysis showed lower‐than‐expected overlap for IAV‐RV A (0.23% vs. 0.40%), IAV‐RV B (0.029% vs. 0.099%), and IAV‐RV A/B/C overall (0.390% vs. 0.624%), while IAV‐RV C was close to random expectation (0.217% vs. 0.210%).

**Conclusion:**

RV and IAV exhibited subtype‐dependent interaction patterns in this regional cohort, with apparent mutual exclusion for RV‐A/RV‐B and near‐random coexistence for RV‐C. These findings support subtype‐stratified surveillance and season‐specific prevention strategies.

## Graphical abstract

This study analyzed 386 rhinovirus infections (dominant Type A, 60.9%; high 73.1% coinfection rate) and Influenza A interactions. Rhinovirus A/B showed mutual exclusivity with Influenza A, while Type C had near‐random association, with temporal prevalence fluctuations observed.

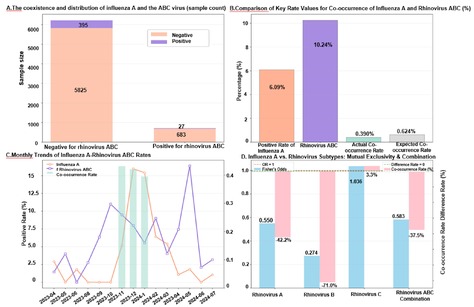

Respiratory virus circulation is shaped by dynamic interactions between pathogens, hosts, and seasonality [1, 2, 3, 4]. Epidemiological evidence from multiple settings suggests that rhinovirus activity may interfere with influenza transmission, but subtype‐specific evidence from Chinese regional cohorts remains limited [5, 6, 7]. Therefore, we conducted a retrospective analysis using routine metagenomic surveillance data to describe RV (A/B/C) epidemiology and to quantify RV‐IAV co‐detection patterns, including observed‐versus‐expected overlap and temporal stage characteristics.

## 1 Materials and Methods

### 1.1 Clinical Data

We retrospectively reviewed respiratory samples tested in Hunan Provincial People's Hospital from April 1, 2023 to December 1, 2025. The full surveillance dataset included 8928 samples. For RV subtype analyses, we applied a predefined quality‐control workflow and established an analytic RV cohort (*n* = 386) by excluding records with incomplete key metadata, repeated records from the same episode, and entries with clinically implausible/background organisms not meeting reporting criteria. Potential selection bias from this workflow is addressed in Section 3 [8, 9].

Demographic variables included age and sex when available. Age groups were predefined as 0–3, 4–12, 13–18, 19–59, and ≥ 60 years. Denominators are explicitly stated for each analysis (overall cohort, RV cohort, IAV cohort, or subgroup denominator).

### 1.2 Pathogen Metagenomic Detection

Pathogens were identified by routine clinical metagenomic next‐generation sequencing under the laboratory's standard operating procedures, including parallel batch controls and standardized quality filtering. Reportable pathogens were interpreted using integrated clinical‐microbiological criteria, and taxonomic names were rechecked against standard nomenclature before statistical aggregation.

### 1.3 Statistical Analysis

Observed co‐occurrence was defined as O12 = n12, and expected co‐occurrence under independence as E12 = (n1 × n2)/N. Categorical associations were tested by Fisher's exact test when any expected cell count was < 5, otherwise chi‐square test. Effect size was summarized with OR and 95% CI. Two‐sided *p* values were used, and Benjamini–Hochberg false discovery rate correction was applied for multiple comparisons across subtype‐level association tests.

## 2 Results

### 2.1 Analysis of the Epidemiological Characteristics of Rhinoviruses

From April 2023 to December 2025, 8928 respiratory samples were reviewed. In the quality‐controlled RV cohort (*n* = 386), subtype A was dominant (235, 60.9%), followed by subtype C (118, 30.6%) and subtype B (33, 8.5%). Mixed‐subtype detection occurred in six samples (1.6%). Age distribution was as follows: 0–3 years, 102 (26.4%); 4–12 years, 89 (23.1%); 13–18 years, 27 (7.0%); 19–59 years, 115 (29.8%); and ≥ 60 years, 53 (13.7%). Detailed demographic and subtype characteristics are summarized in Table 1, and the coinfection profile is summarized in Table 2.

**TABLE 1:** Characteristics of rhinovirus infection.

Essential information	Specific values/proportions	Detailed data/notes
**Analytic rhinovirus cohort size**	386 cases	
Number of Type A infections/proportion	235 cases/60.9%	Dominant subtype
Number of Type C infections/proportion	118 cases/30.6%	Subdominant subtype
Number of Type B infections/proportion	33 cases/8.5%	The lowest infection subtype
**Mixed subtype**	6 cases/1.6%	
A + C type mixture	4 cases/1.04% (of RV cohort)	Accounting for 66.7% of the mixed subtypes
A + B type mixture	1 case/0.26% (of RV cohort)	Accounting for 16.7% of the mixed subtypes
B + C type mixture	1 case/0.26% (of RV cohort)	Accounting for 16.7% of the mixed subtypes
**Age distribution**		
**0–3 years old**	102 cases/26.4%	One of the high‐risk groups
Percentage of Type A (within the group)	33 cases/32.3%	
Percentage of Type C (within the group)	36 cases/35.2%	
Percentage of Type B (within the group)	33 cases/32.5%	
**4–12 years old**	89 cases/23.1%	
Percentage of Type A (within the group)	55 cases/61.8%	
Percentage of Type C (within the group)	28 cases/31.5%	
Percentage of Type B (within the group)	6 cases/6.7%	
**13–18 years old**	27 cases/7.0%	
Percentage of Type A (within the group)	17 cases/63%	
Percentage of Type C (within the group)	8 cases/29.6%	
Percentage of Type B (within the group)	2 cases/7.4%	
**19–59 years old**	115 cases/29.8%	One of the high‐risk groups (with the highest proportion)
Percentage of Type A (within the group)	75 cases/65.2%	
Percentage of Type C (within the group)	32 cases/27.8%	
Percentage of Type B (within the group)	8 cases/7%	
**Over 60 years old**	53 cases/13.7%	
Percentage of Type A (within the group)	31 cases/58.5%	
Percentage of Type C (within the group)	14 cases/26.4%	
Percentage of Type B (within the group)	8 cases/15.1%	

**TABLE 2:** Statistics on coinfection with rhinovirus and other pathogens.

Concurrent infection	Specific values/proportions	Note
**Number of simple rhinovirus infections/proportion**	104 cases/26.9%	
**Number of concurrent infections/proportion**	282 cases/73.1%	The rate of coinfection is extremely high
**Bacterial coinfection (overall)**	179 cases/63.5%	The most common type of combination
The number of *Streptococcus pneumoniae* infections	58 cases	Bacteria rank first
The number of *Haemophilus influenzae* infections	47 cases	
The number of *Klebsiella pneumoniae* infections	42 cases	
The number of *Pseudomonas aeruginosa* infections	39 cases	
Number of *Staphylococcus aureus* infections	23 cases	
**Viral coinfection (overall)**	126 cases/44.7%	
Human herpesvirus type 5 (CMV)	68 cases	Top of the list is the virus category
Number of respiratory syncytial virus infections	29 cases	
The number of COVID‐19 infections	17 cases	
Number of influenza A virus infections	14 cases	
The number of parainfluenza virus infections	12 cases	
**Fungal/mycoplasma/chlamydia coinfection**		
The number of *Mycoplasma pneumoniae* infections	53 cases	
The number of *Pneumocystis jirovecii* detections	36 cases	
Number of *Candida albicans* infections	28 cases	
Parrot fever/ *Chlamydia pneumoniae* infection count	19 cases	
**Multiple coinfections (bacteria + virus + fungi/mycoplasma)**	32 cases/11.3%	Bacteria + viruses + fungi/mycoplasma
The number of infections in the infant group	14 cases/43.8%	
The number of infections in the elderly group	10 cases/31.3%	

### 2.2 Analysis of the Epidemiological Characteristics of Influenza A Virus

A total of 422 IAV‐positive samples were identified. Single IAV detection accounted for 52/422 (12.32%), whereas coinfection accounted for 370/422 (87.68%). The most frequent bacterial, fungal, and viral copathogens are summarized in Table 3 after nomenclature standardization.

**TABLE 3:** Prevalence of influenza A virus.

Infection type	Specific values/proportions	Detection times
**Single influenza A infection**	52 cases/12.32%	
**Coinfection**	370 cases/87.68%	
**Co‐infectious pathogens of bacteria**	*Pseudomonas aeruginosa*	76
	*Klebsiella pneumoniae*	68
	*Streptococcus pneumoniae*	59
	*Haemophilus influenzae*	52
	*Staphylococcus aureus*	45
	*Acinetobacter baumannii*	41
	*Stenotrophomonas maltophilia*	18
	*Enterobacter cloacae* complex group	12
	*Elizabethkingia anophelis*	8
**Fungal coinfection pathogens**	*Aspergillus fumigatus*	63
	*Candida albicans*	35
	*Aspergillus flavus*/*Aspergillus oryzae*	31
	*Pneumocystis jirovecii*	29
	*Aspergillus nidulans*	17
	*Rhizopus microspora*	14
	*Candida glabrata*	11
	*Candida tropicalis*	9
	*Aspergillus niger*	7
	*Lichtheimia corymbifera*	3
**Viral coinfection pathogens**	Human herpesvirus type 1	58
	Human herpesvirus type 5 (CMV)	51
	Human coronavirus 229E	24
	SARS‐CoV‐2	11
	Rhinovirus (types A/B/C)	36
	Human herpesvirus type 7	19
	Human adenovirus group B	13
	Human respiratory syncytial virus	10
	Human parainfluenza virus	8

### 2.3 Analysis Results of the Mutual Exclusion Between Influenza A and Rhinovirus A

#### 2.3.1 Mutual‐Exclusion Pattern Between IAV and RV‐A

RV‐A showed a lower‐than‐expected overlap with IAV. The observed co‐occurrence rate (0.23%) was below the expected rate under independence (0.40%), supporting a negative association at the population level.

**FIGURE 1:**
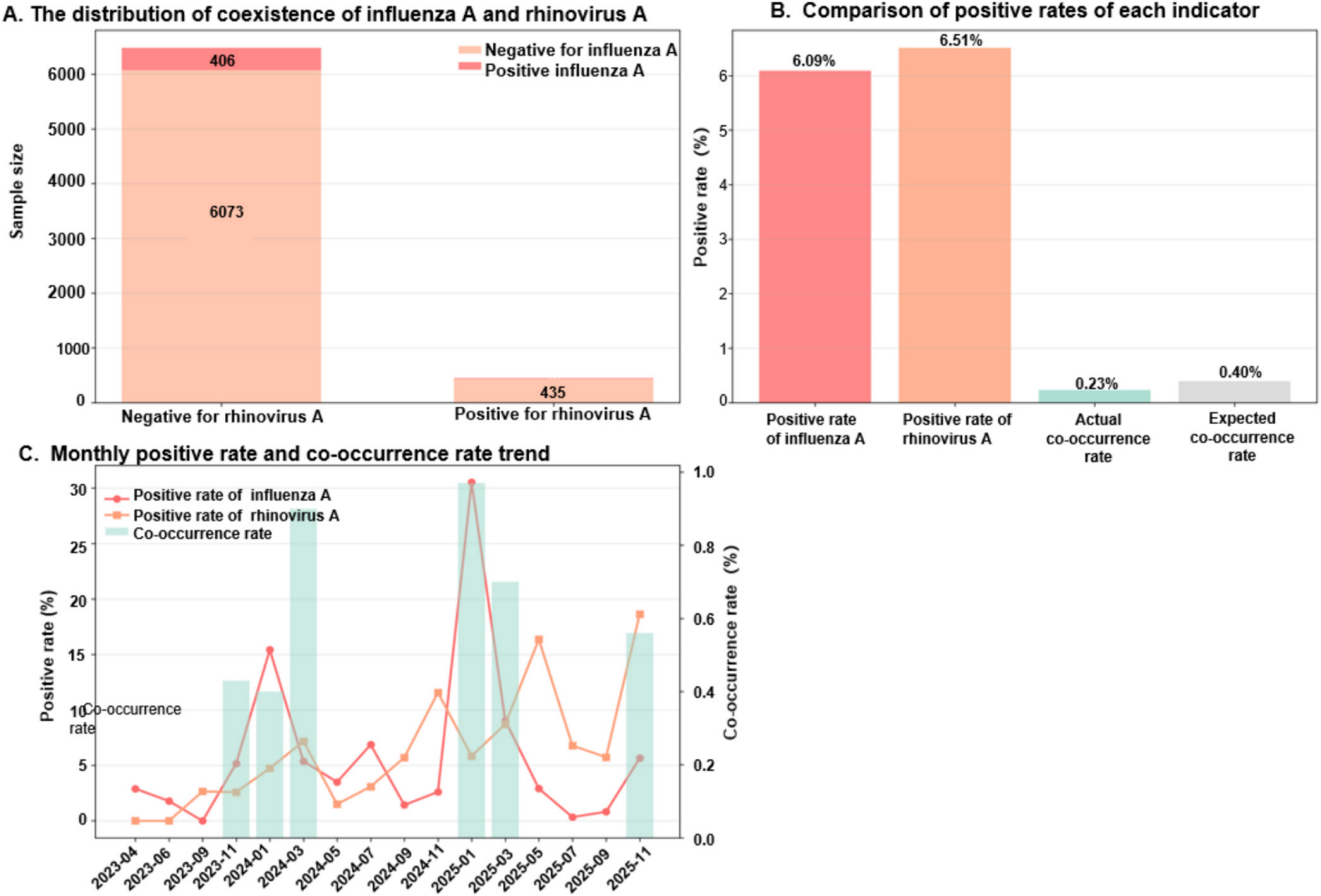
Analysis of the Exclusivity between Influenza A and Rhinovirus A.

Figure 1 summarizes subtype‐level prevalence, observed‐versus‐expected co‐occurrence, and monthly trend trajectories. IAV and RV‐A did not show synchronized high‐prevalence periods, and their overlap remained low throughout the observation window.

### 2.4 Analysis Results of the Co‐Occurrence Distribution and Epidemic Characteristics of Influenza A and Rhinovirus B

#### 2.4.1 Association Pattern Between IAV and RV‐B

For RV‐B, the observed co‐occurrence rate with IAV (0.029%) was substantially lower than expected (0.099%), consistent with a stronger mutual‐exclusion tendency than RV‐A.

Temporal curves showed modest RV‐B fluctuations with limited overlap during IAV peaks, supporting a persistent negative association rather than random superposition.

**FIGURE 2:**
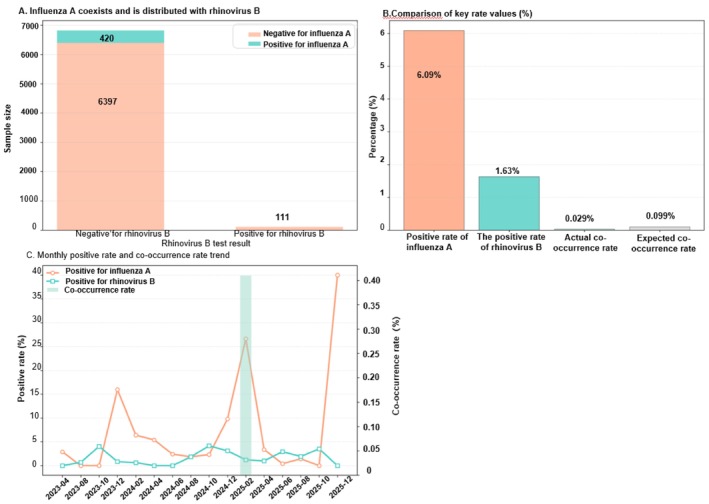
Co‐occurrence, prevalence, and temporal trends of influenza A and rhinovirus B.

Note: Figure 2 presents prevalence indicators and temporal co‐occurrence trends for IAV and RV‐B. The effect‐size direction was consistent with a mutually exclusive pattern.

### 2.5 Co‐Occurrence and Epidemiological Characteristics Analysis Results of Influenza A and Rhinovirus C

#### 2.5.1 Association Pattern Between IAV and RV‐C

In contrast to RV‐A and RV‐B, RV‐C showed near‐random co‐occurrence with IAV. The observed rate (0.217%) was close to the expected rate (0.210%), indicating no clear exclusion or synergistic pattern in this cohort.

**FIGURE 3:**
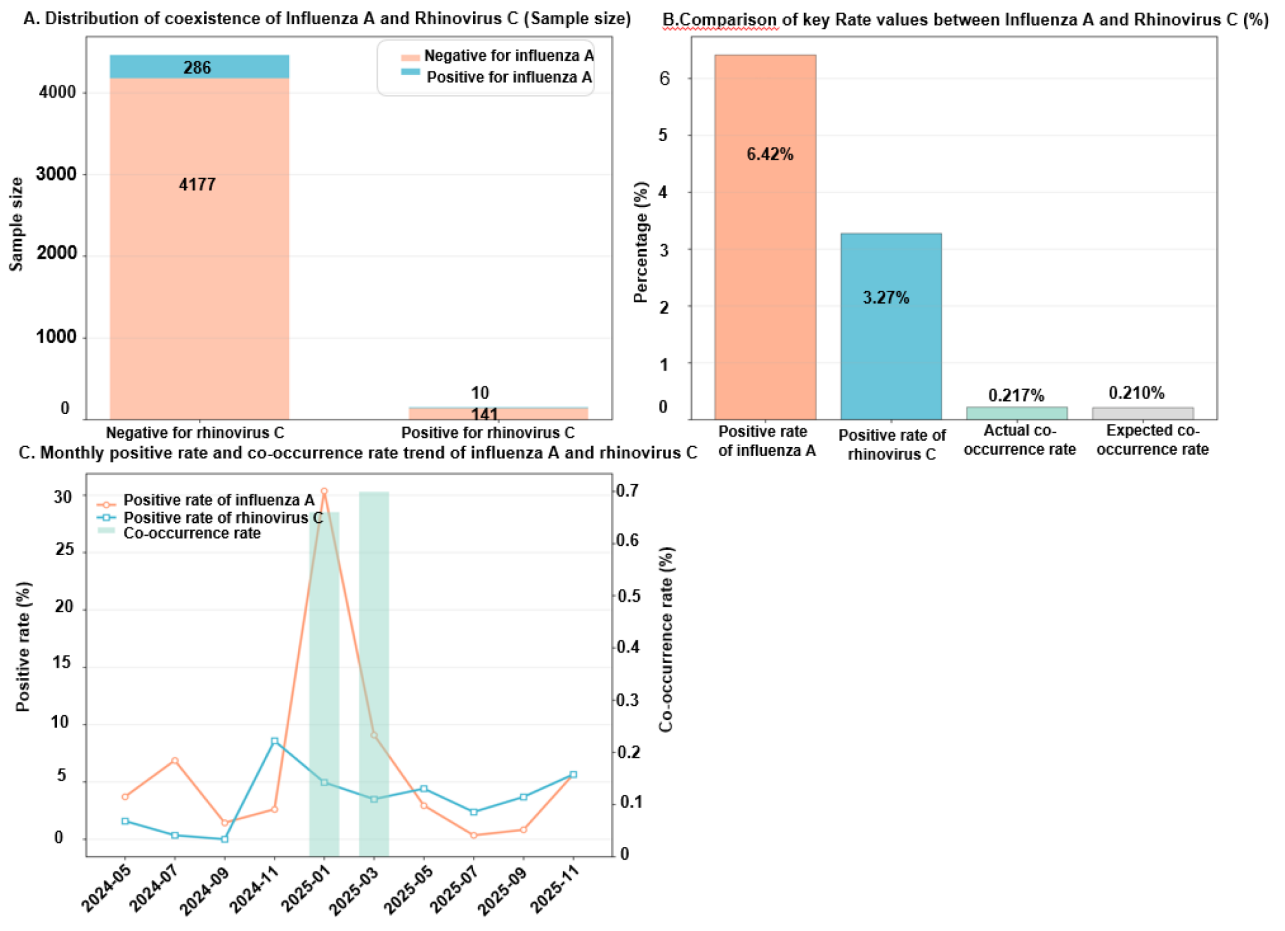
Co‐occurrence distribution, prevalence, and temporal trends of influenza A and rhinovirus C.

Note: Figure 3 shows that RV‐C and IAV occasionally rose in parallel in epidemic periods; however, the observed‐versus‐expected comparison remained compatible with random overlap.

### 2.6 Co‐Occurrence Characteristics, Prevalence, and Subtype Mutual‐Exclusion Analysis of Influenza A and ABC Rhinovirus

#### 2.6.1 Integrated Analysis of IAV and RV‐A/B/C

At the combined subtype level, observed IAV‐RVABC co‐occurrence (0.390%) remained lower than expected (0.624%), indicating an overall negative association. Subtype‐stratified OR patterns were directionally consistent with stronger exclusion for RV‐A and RV‐B, while RV‐C was close to neutral.

**FIGURE 4:**
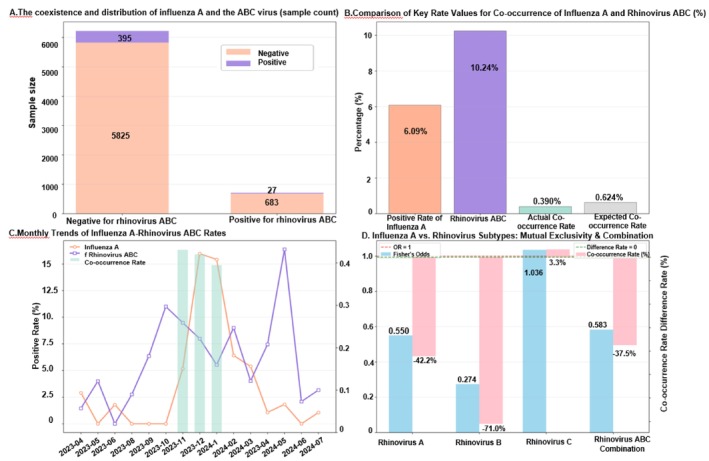
Co‐occurrence distribution, prevalence, and subtype mutual exclusivity of influenza A and rhinovirus ABC.

Note: Figure 4 provides integrated prevalence, co‐occurrence, and subtype‐level association comparisons. Denominators are panel‐specific and are reported in the figure labels.

### 2.7 Analysis of the Epidemic Trends and Stage Correlations Between Influenza A and Three Rhinoviruses

To describe long‐term dynamics, we examined staged trends from 2020 to 2025. A four‐stage pattern was observed: low‐activity baseline, synchronized activation, stepwise amplification, and high‐fluctuation plateau. Across stages, IAV remained the dominant seasonal driver, while RV subtypes showed subtype‐dependent coupling intensity.

**FIGURE 5:**
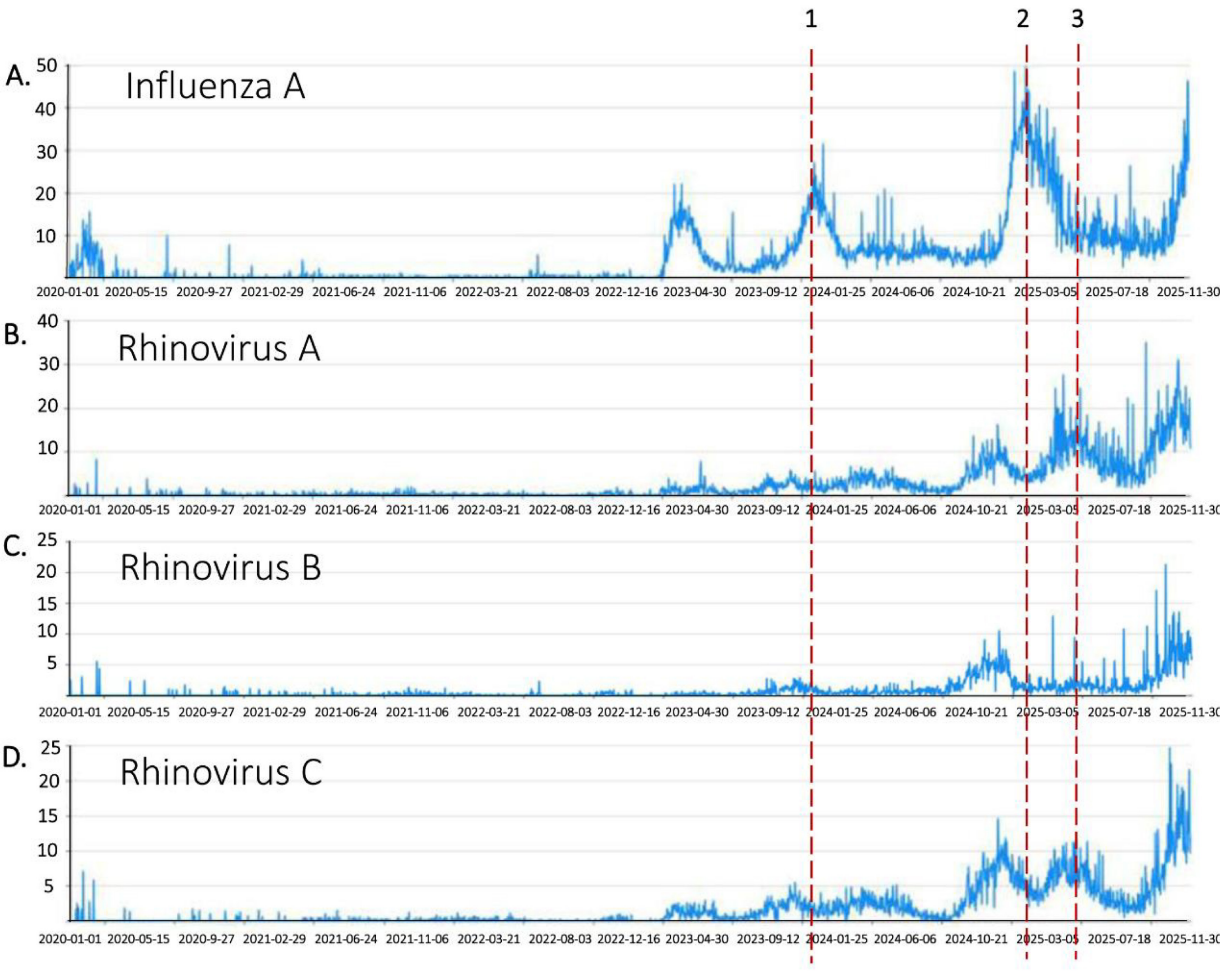
Fluctuation trends of influenza A and rhinovirus (A/B/C) prevalence from 2020 to 2025.

Note: Figure 5 displays stage boundaries and subtype‐specific monthly prevalence trajectories. The figure is intended for epidemiological pattern interpretation rather than mechanistic inference.

## 3 Discussion

This study provides regional surveillance evidence on RV‐IAV interaction patterns using routine clinical metagenomic data [10, 11, 12, 13, 14]. Three findings are robust in the revised analysis: (1) RV‐A and RV‐B showed lower‐than‐expected co‐occurrence with IAV [15, 16, 17]; (2) RV‐C showed near‐random overlap [18, 19, 20, 21]; and (3) RV coinfection burden was high, with substantial bacterial and viral copathogen detection [22, 23, 24, 25].

We intentionally toned down mechanistic claims. The observed subtype‐specific differences are epidemiological associations and should not be interpreted as direct biological causation without functional validation. Mutation‐level and antiviral‐resistance inferences not directly supported by this dataset were removed or reframed as literature‐supported possibilities [26, 27].

From a public‐health perspective, these data support subtype‐stratified surveillance and stage‐adaptive prevention strategies [28]. During periods of high IAV activity, concurrent monitoring of RV subtypes and key copathogens may improve risk stratification and resource allocation in respiratory care settings [29].

Limitations include the retrospective single‐center design, incomplete covariates for some patients (for example, underlying disease and immune status), and the absence of direct experimental validation of interference mechanisms. Multicenter prospective studies integrating viral load and host‐response markers are needed to confirm causality [30, 31].

## Author Contributions

Feicheng Yang drafted the manuscript and coordinated revisions. Ping Bai, Jiaxuan Zhang, and Juqi Peng curated data and performed preliminary analyses. Qing Hu and Yanchun Li supervised study design, interpretation, and final manuscript approval. All listed authors contributed substantially and approved the final version.

## Funding

This work was supported by the Hunan Provincial Natural Science Foundation (Nos. 2025JJ60591, 2026JJ81728) and the Young Doctoral Fund Project of Hunan Provincial People's Hospital (also known as the 2023 National Natural Science Foundation Cultivation Project) (No. BSJJ202218). The authors would like to thank the funding agencies for their financial support.

## Ethics Statement

This retrospective study was approved by the Ethics Committee of Hunan Provincial People's Hospital (The First Affiliated Hospital of Hunan Normal University) (Approval No. ky2025‐138). The requirement for informed consent was waived due to anonymized data use and minimal‐risk design.

## Conflicts of Interest

The authors declare no conflicts of interest.

## Data Availability

De‐identified data supporting the findings are available from the corresponding authors upon reasonable request and institutional approval.
